# Rapid autofluorescence flow cytometric analysis of agonist-induced neutrophil and eosinophil polarization reveals novel insights into 5-oxo-ETE-mediated granulocyte activation

**DOI:** 10.1186/s12950-025-00472-8

**Published:** 2025-11-06

**Authors:** Anuruddika J. Fernando, Fiona Rossi, Destiny Docherty, Anna Popravko, Lucy Masters, Boydd Houston, Renu Gupta, Kevin Dhaliwal, Adriano G. Rossi

**Affiliations:** 1 Centre for Inflammation Research, Institute for Regeneration and Repair, University of Edinburgh, 5 Little France Drive, Edinburgh, Scotland EH16 4UU UK; 2 Flow Cytometry & Cell Sorting Facility, Institute for Regeneration and Repair, University of Edinburgh, 5 Little France Drive, Edinburgh, Scotland EH16 4UU UK; 3 Adiso Therapeutics, Concord, MA USA

**Keywords:** Granulocytes, Neutrophils, Eosinophils, Shape-change, Flow-cytometry, 5-oxo-ETE

## Abstract

Minimizing unintended granulocyte activation while measuring functional responsiveness is essential, as the use of external probes, antibodies, or fluorescent dyes can potentially alter cellular responsiveness. To address this, we employed an antibody-free flow cytometry approach that measures forward scatter (FSC) to detect variations in cell-size, morphology, and shape; some key indicators of neutrophil and eosinophil activation. Human peripheral blood neutrophils, containing contaminating eosinophils, were isolated using discontinuous Percoll gradients and pre-treated with receptor antagonists [e.g., cyclosporin-H (an FPR1 antagonist) and CP105696 (a BLT1 receptor antagonist)] prior to stimulation with agonists such as fMLF (an FPR1 agonist) and LTB_4_ (a BLT1 agonist). Furthermore, fMLF stimulation resulted in a loss of CD62L and an increase in CD11b expression along with an increase in intracellular ROS production compared to control, as analysed using flow cytometry. Imaging flow cytometry, together with FSC analysis, enabled assessment of cell polarization and associated morphological changes. Importantly, autofluorescence-based gating allowed for the identification of contaminating eosinophils within the mixed granulocyte population, allowing parallel assessment of shape-change in both neutrophils and eosinophils in response to the same ligands. Stimulation of neutrophils with fMLF resulted in distinct FSC shifts compared to unstimulated controls across all flow cytometers tested, which were inhibited by cyclosporin-H, but not CP105696. Morphological analysis confirmed these changes corresponded with increased cell area and perimeter and decreased circularity, hallmarks of cell polarisation. Additionally, selective activation of eosinophils (but not neutrophils) by eotaxin, and dual activation of both cell types by the arachidonic acid metabolite 5-oxo-ETE, were confirmed through specific gating strategies. Taken together, these findings support the use of FSC-based flow cytometry as a rapid, scalable and effective method for evaluating granulocyte polarisation and screening candidate therapeutics targeting immune cell activation in disease contexts.

**Supplementary Information:**

The online version contains supplementary material available at 10.1186/s12950-025-00472-8.

## Introduction

Granulocytes, primarily neutrophils and eosinophils, are key bone-marrow derived inflammatory cells of the innate immune system and neutrophils, as first responders, are rapidly recruited to sites of infection and tissue injury [1–3]. These immune cells are guided to inflammatory sites by various chemoattractants, including formylated peptides such as N-formylmethionine-leucyl-phenylalanine (fMLF), a potent synthetic peptide agonist which mimics bacterial infection or mitochondrial damage, is known to ligate FPR1 to activate neutrophils [4]. Similarly, the arachidonic-acid derived mediators like leukotriene B_4_ (LTB_4_) and 5-oxo-ETE, and the cytokine CXCL8 (also known as IL-8) also activate downstream neutrophil functions [1, 5, 6]. These agonists act through binding to specific G-protein coupled receptors (GPCRs); fMLF activates FPR1, LTB_4_ binds BLT1, 5-oxo-ETE signals via OXER1, and eotaxin binds CCR3 primarily expressed on eosinophils [4, 7–10]. Physiologically, granulocyte priming is considered a necessary step for full activation, facilitating receptor expression, degranulation and production of reactive oxygen species (ROS) [11]. Upon activation, neutrophils and eosinophils initiate key effector functions evolved at neutralising pathogens and resolving tissue damage. However, dysregulated or uncontrolled granulocyte activation can contribute to chronic inflammation and collateral tissue damage [4, 5]. Therefore, the possibility of screening pharmaceutical compounds such as receptor antagonists to counteract this activation, could be therapeutically beneficial.

Many in vitro assays have been developed to assess downstream granulocyte responses, such as intracellular calcium flux, chemotaxis, degranulation, phagocytosis, ROS generation, and extracellular trap formation. However, these methods often require the use of fluorogenic dyes [(e.g., dihydrorhodamine (DHR) that detects intracellular ROS)], fluorescent antibodies and chemiluminescent probes (e.g., lucigenin detects superoxide anions), which can penetrate cells and may themselves inadvertently activate granulocytes and skew results [12–14]. Furthermore, given how readily granulocytes can be activated, the isolation procedures may themselves stimulate cells, potentially affecting their downstream functions [15, 16]. Use of a well-established method combining dextran sedimentation with discontinuous Percoll gradient centrifugation offers high granulocyte purity (≥ 95%), similar to negative selection methods using immunomagnetic beads, while minimising activation of immune cells [17, 18]. However, minimal contamination with highly autofluorescent eosinophils in neutrophil preparations has allowed for the distinction between these two cell types using flow cytometry, enabling their use in various applications [19–21].

In this study, we present a simple and reliable antibody-free flow cytometry method for detecting neutrophil and eosinophil activation based on their scatter profiles [forward scatter (FSC) and side scatter (SSC)], which reflect changes in cell size, shape, and granularity. In addition, this technique is compatible with both standard flow cytometers and imaging flow cytometers, offering the added benefit of visualising cell morphology at the single-cell level following activation [22, 23]. Although similar approaches have been used in other studies to assess neutrophil activation, we expand on this by incorporating imaging flow cytometry and applying the method to explore previously uncharacterised mechanisms of neutrophil and eosinophil activation, in response to agonists such as eotaxin and 5-oxo-ETE [24, 25].

Using this approach, we examined shape changes (quantified by FSC-A; forward scatter-area) in neutrophils and eosinophils in response to specific GPCR agonists (e.g., fMLF and LTB_4_) and their respective antagonists [e.g., cyclosporin-H (CsH) and CP105696]. Although previous studies have examined the role of LTB_4_ and its receptor antagonist in neutrophil shape change and apoptosis [26], comparative analyses with other receptor agonists and antagonists, such as CsH, have not been reported. Our study addresses this gap by directly comparing these effects and moreover, while morphological changes in primed and activated neutrophils have been described, we employ imaging flow cytometry to visualise these changes in real-time, offering new insights into the dynamic behaviour of neutrophils with detail not previously achieved [27]. We further analysed the differential effects of 5-oxo-ETE and the chemokine eotaxin on neutrophil and eosinophil cellular polarisation. Multiple analysis strategies were used to assess granulocyte shape change, providing evidence into a rapid and scalable method for screening immune cell responses and identifying potential therapeutic targets.

## Methods

### Ethics statement

Human blood samples were collected from healthy adult volunteers with informed consent, in accordance with ethical approval granted by the Lothian Research Ethics Committee (EMREC Reference: 21-EMREC-041). All collections were carried out at the Institute for Regeneration and Repair (IRR; University of Edinburgh) by registered phlebotomists, adhering to established institutional and local guidelines.

### Isolation of human granulocytes from peripheral blood

Peripheral human blood was collected into Falcon tubes containing 3.8% sodium citrate (Sigma). Leukocytes were isolated using dextran (Sigma) sedimentation followed by discontinuous Percoll (GE Healthcare) gradient centrifugation. Mononuclear cells were collected from 55/70% interface, while granulocytes were isolated from the 70/81% interface, as described [14, 20]. The resulting granulocytes consisting primarily of neutrophils with some (usually less than 5%) eosinophils, were washed with phosphate-buffered saline (PBS) without cations and cell counts were performed using a haemocytometer. Cell purity was assessed by cytocentrifuge preparations, with ≥ 95% of cells identified as neutrophils with minor eosinophil contamination. Cells were resuspended in PBS with cations (Mg^2+^ and Ca^2+^) at the appropriate concentration for downstream assays.

### Assessment of neutrophil purity

To assess cell morphology and determine neutrophil purity, cytocentrifuge preparations were carried out using 1 × 10^6^ granulocytes in 100µL of suspension. Cells were loaded onto cytocentrifuge chambers and centrifuged at 300 rpm for 3 min using a Thermo Scientific Shandon cytocentrifuge. The resulting cell films were briefly air-dried, fixed in 100% methanol, and subsequently stained with Eosin and Haematoxylin using Diff-Kwik solutions 2 and 3 (Thermo Scientific), respectively. Slides were mounted with DPX mounting medium (Sigma) and examined under a light microscope at 100 × magnification. Neutrophil purity was determined by counting at least 100 cells across 10 randomly chosen fields of view, based on standard morphological criteria (e.g., number of nuclear lobes and staining characteristics of the cytoplasm and granules).

### Granulocyte cell polarisation (shape change assay)

To assess the effects of receptor agonists and antagonists on neutrophil activation by measuring shape change, isolated human granulocytes were resuspended at 2 × 10^6^ cells/mL. Cells were pre-incubated with the antagonists, CsH (Sigma) or CP105696 (MedchemExpress), or PBS vehicle control, for 30 min at 37 °C on a shaking heat block (300 rpm). Antagonist concentrations used are provided in the results section or figure legends. Following antagonist incubation, cells were stimulated with receptor-specific agonists; fMLF (Sigma) or LTB_4_ (Cayman chemical) for 30 min, and eotaxin (Peprotech) or 5-oxo-ETE (Avanti polar lipids) for 2 or 5 min at 37 °C, based on prior concentration–response and time-course optimisation experiments. Post stimulation, cells were fixed by adding an equal volume of 4% paraformaldehyde (PFA, ChemCruz) in PBS and stored at 4 °C until analysis. Neutrophil shape change was assessed by measuring changes in forward scatter (FSC-A) using flow cytometry analysers and sorters such as the BD LSR Fortessa (BD Biosciences) and BD FACS Aria II (BD Biosciences), respectively. Data were analysed with FCSExpress 7 (research edition), and shape-change was quantified based on forward scatter area (FSC-A), including area under the curve derived from histogram width and weight, as previously described [28].

For comparison and standardisation, activation data were re-analysed using FCSExpress 7 software. A marker was applied at the same position on the y-axis for both control and stimulated histograms to define a region of interest. Using the statistics function, the percentage of total cells was determined, representing the area under the curve for the selected region.

### Imaging flow cytometry

In addition to assessing neutrophil activation based on light scatter parameters, isolated neutrophils treated with fMLF, or PBS vehicle control were analysed using imaging flow cytometry on the Thermo Attune Cytpix system (Thermo Scientific). This platform integrates traditional flow cytometry with high-resolution imaging, at the single cell level, enabling detailed morphological assessment. Image analysis was performed on single cells using Attune cytometric software, which quantified neutrophil phenotypic parameters such as circularity, cell area, and perimeter (in microns), in both activated and non-activated neutrophils.

Due to inherent autofluorescence of eosinophils, these cells were distinguished from neutrophils using autofluorescence-based gating, as described [20]. Granulocytes were first gated based on forward and side scatter profiles with doublets excluded using FSC-area and FSC-height parameters. Human eosinophils were differentiated from neutrophils using the 488 nm laser (525/50 nm filter), in combination with either conventional side scatter profiles or signals from the 355 nm laser (450/50 nm filter). This gating strategy enabled identification of both cell types within a single plot, allowing quantification of shape-change responses to various agonists and antagonists in neutrophils and eosinophils.

### Cell surface marker expression

For cell surface marker analysis, neutrophils treated with 100 nM fMLF or control PBS were incubated for 20 min at 4 °C in the dark with CD62L-PE/Cy7 (Invitrogen, 25–0629-42), CD11b-AF488, clone ICRF44 (Biolegend, 301,318), and Trustain FcX human Fc block (Biolegend, 422302). Cells were then centrifuged at 300 × g for 5 min, resuspended in 300µL of 1% BSA in PBS, and stained with DAPI (1:1000) before flow-cytometric analysis (ACEA Novocyte).

### Intracellular reactive oxygen species (ROS) generation

The effect of agonists such as fMLF on ROS production in human neutrophils was assessed using dihydrorhodamine-1,2,3 (DHR-1,2,3; Sigma). Neutrophils isolated from healthy donor blood were resuspended in HBSS containing with divalent cations at 10 × 10^6^ cells/mL. A 600µL aliquot of this suspension was diluted in 4.8 mL HBSS media and incubated with 600µL of 10 µM DHR 1,2,3, resulting in a final concentration of 1 × 10^6^ cells/mL with 1 µM DHR. Cells were incubated for 5 min at 37 °C in a waterbath, and 240µL aliquots were transferred to 2 mL Eppendorf tubes. Each aliquot was then treated with 30µL of diluted fMLF and PBS, followed by incubation for 30 min at 37 °C on a benchtop heat block with shaking (300 rpm). Intracellular ROS was quantified by measuring fluorescence at 488 nm were using flow cytometry (Acea Novocyte), with or without fixation in 4% PFA/PBS.

### Statistical analyses

All data (unless specified) are presented as mean ± standard error of the mean (SEM) based on experimental replicates from individual donors. Flow-cytometry data were analysed using FCSExpress7 (research edition) and visualised using GraphPad Prism (version 9). Statistical significance was determined using one-way ANOVA followed by Tukey’s post hoc multiple-comparisons test for analyses involving more than two treatment groups. Comparisons related to neutrophil image analysis and shape change measurements from different flow cytometry instruments were assessed using unpaired Student’s *t*-tests. Statistical significance was interpreted as *p* ≤ 0.05 (*), *p* ≤ 0.01 (**), *p* ≤ 0.001 (***) and *p* ≤ 0.0001 (****).

## Results

### Integrated conventional and imaging flow cytometry provide complementary insights into neutrophil activation, inhibition, and morphological dynamics

**Fig. 1:**
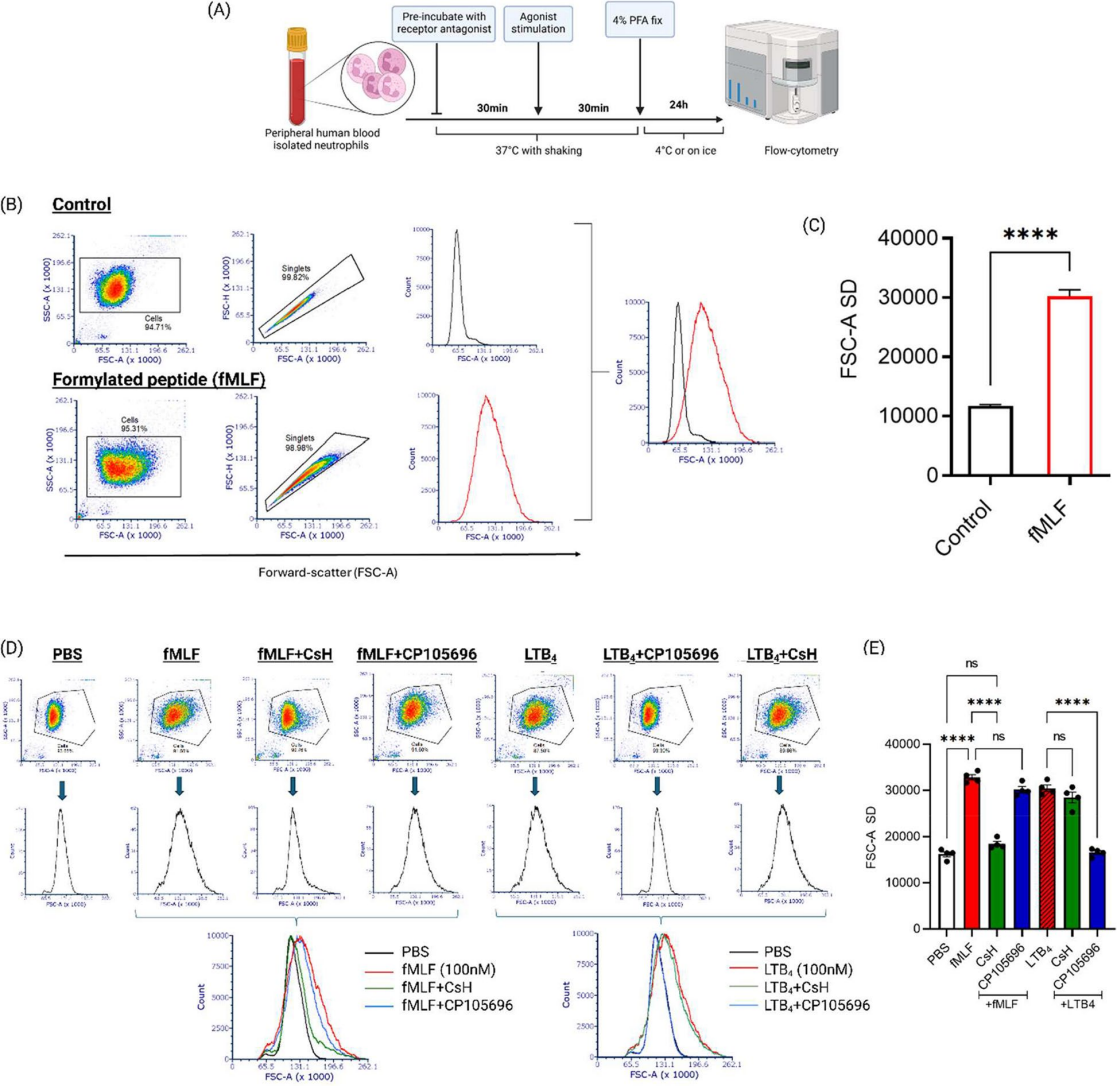
fMLF and LTB_4_ trigger human neutrophil shape change, detected by FSC-A and cell morphological alterations. **A** Schematic of the human neutrophil shape change assay. Neutrophils were isolated by dextran sedimentation followed by Percoll gradient centrifugation, then incubated with PBS or receptor antagonists prior to agonist stimulation (e.g., fMLF). Cells were analysed following fixation by flow cytometry. **B** Gating strategy and representative histogram plots showing no activation following PBS treatment, while fMLF stimulation induced shape changes detectable as shifts in forward-scatter (FSC-A) on a BD FACSAria II. **C** Quantification of human neutrophil shape change in response to fMLF compared to PBS across ten independent donor experiments (*n* = 10). **D** Flow cytometry histograms and overlays demonstrating receptor-specific inhibition of neutrophil shape change (e.g., CP105696 inhibits LTB_4_ -mediated activation), indicating blockade of agonist-induced responses. **E** fMLF-induced shape change (100 nM) was inhibited by CsH (10 µM), while CP105696 (10 µM) blocked LTB_4_ (100 nM)-induced shape change but not fMLF-induced responses, confirming specific inhibition of FPR1 and BLT1 receptors. ****p ≤ 0.0001, unpaired t-test with Welch’s correction (PBS vs. fMLF; Fig. 1 C); one-way ANOVA with Tukey’s post hoc test for multiple comparisons between treatment groups (Fig. 1E)

Shape-changes in response to chemoattractants allow neutrophils to migrate and usually squeeze in between endothelial cells during transmigration to inflammatory sites. To establish and quantify this response, we assessed neutrophil shape change in response to chemoattractants using forward scatter area (FSC-A) as a readout for neutrophil activation. Agonist and antagonist concentrations, as well as incubation times, were optimised through concentration–response and time-course experiments (Supplementary Figs. 1 and 2) and guided by existing literature [26, 29, 30]. Neutrophils isolated from healthy human blood using dextran sedimentation and Percoll gradients were pre-treated with GPCR antagonists such as cyclosporin-H (CsH) and CP10569, prior to agonist stimulation, followed by fixing and analysis using flow cytometry (Fig. 1A).

Stimulation of neutrophils with 100 nM fMLF resulted in a significant increase in shape-change compared to unstimulated controls, as detected by an increase in forward scatter area (FSC-A) following gating for granulocytes and singlet populations using a BD FACS Aria II cell sorter (*p* < 0.05, *n* = 10; Fig. 1B&C). In addition, side scatter was monitored in all experiments, but only minimal, donor-dependent variations in granularity were observed. The lack of consistent SSC shifts suggested that the cells did not undergo degranulation. As SSC proved less informative in this context, our analysis focused primarily on FSC, which more directly reflects changes in cell size and shape.

The same approach was used to assess inhibition of neutrophil activation using GPCR antagonists. Cyclosporin-H and CP105696 alone did not induce neutrophil shape-change (data not shown), but each selectively inhibited the corresponding receptor-mediated response. CsH significantly reduced fMLF-induced neutrophil shape change but had no effect on LTB_4_-induced activation, whereas CP105696 significantly inhibited LTB_4_-mediated shape change but did not affect fMLF-induced response (Fig. 1D&E). These results highlight the use of this method in studying both receptor-specific activation and inhibition of neutrophils using defined ligands. Furthermore, although fMLF can result in secondary LTB_4_ production, the lack of effect of CP105696 on fMLF-induced shape change confirms that this response is not mediated via the LTB_4_/BLT1 pathway [26, 31, 32].

**Fig. 2:**
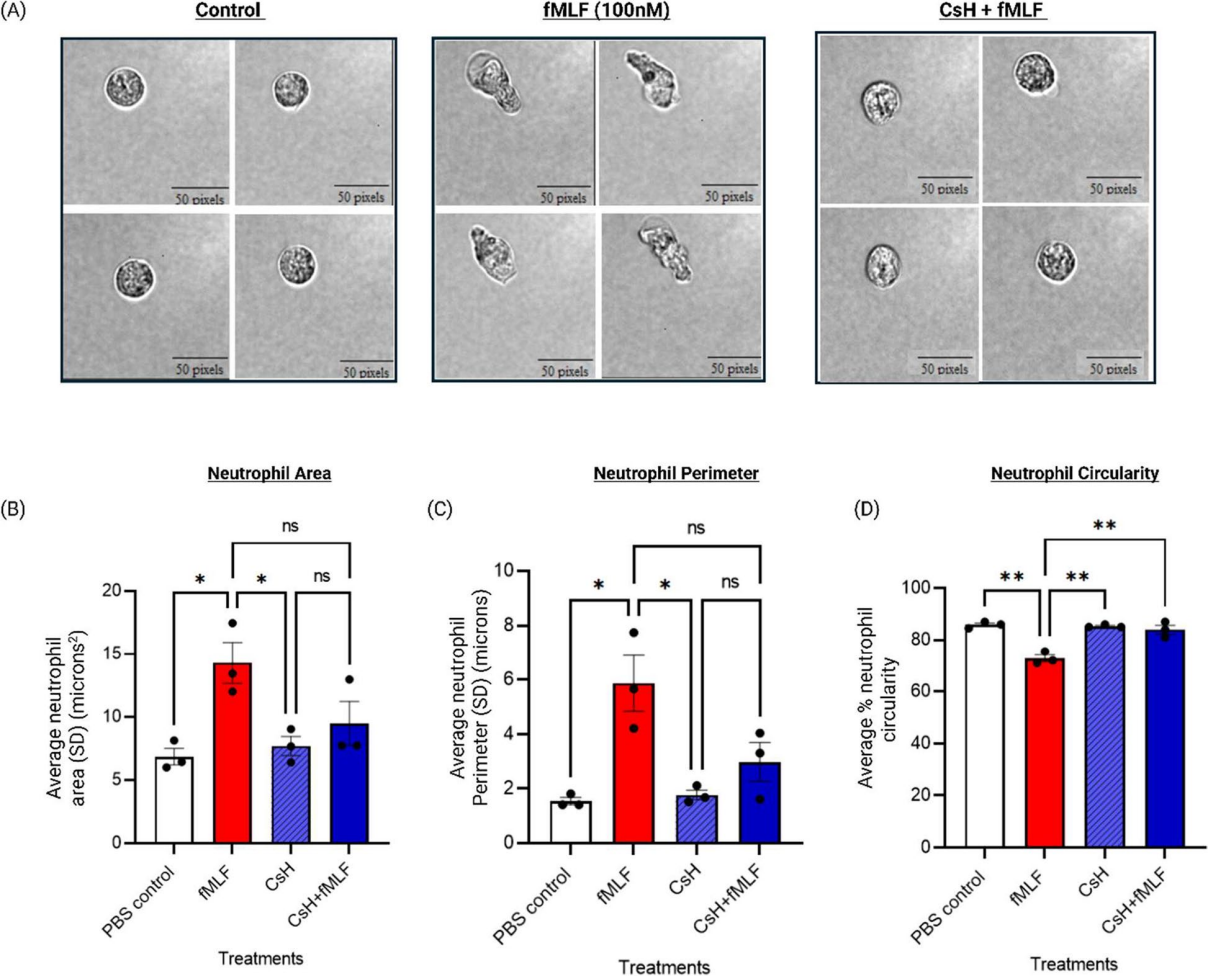
Neutrophil activation induces morphological changes detectable by flow cytometry, which were inhibited by CsH treatment. Imaging flow cytometry was used to assess changes in cell shape and size during neutrophil activation, alongside forward scatter analysis. **A** Representative brightfield images showing neutrophil elongation and irregular morphology following stimulation with fMLF (100 nM), which was inhibited by CsH (10 µM), reverting to a morphology similar to unstimulated controls. **B**-**D** Quantitative morphological analysis using the Attune Cytpix imaging flow cytometer and associated software, revealed that fMLF stimulation increased neutrophil area and perimeter, while reducing circularity. These changes were inhibited by CsH treatment. n ≥ 100 cells per condition were analysed across 3 independent experiments. *p ≤ 0.05, **p ≤ 0.01; one way ANOVA with Tukey’s post hoc test. Data are presented as Mean ± SEM

Developments in flow-cytometry have enabled real-time visualization of individual cells and assessment of morphological features alongside fluorescence-based measurements [25]. To validate neutrophil shape changes observed by forward scatter (FSC-A), we used the Attune Cytpix imaging flow cytometer to directly image neutrophils following stimulation with fMLF or PBS, and in the presence of cyclosporin-H. Using the Attune Cytometric software (v6.21), hundreds of cells per sample were analysed. Unstimulated neutrophils exhibited a rounded morphology, while fMLF-stimulated cells displayed elongated and irregular shapes consistent with activation-induced morphological changes (Fig. 2A). Neutrophil morphological parameters such as circularity, perimeter, and area were quantified in cells from three donors under unstimulated conditions, following fMLF stimulation, and after pre-incubation with CsH. fMLF stimulation significantly increased cell perimeter and area, while reducing circularity, consistent with forward scatter findings (Fig. 2B-D). Pre-treatment with CsH resulted in a significant increase in cell circularity, accompanied by reductions in both area and perimeter, restoring neutrophil shape to control-like levels. In addition, CsH alone did not cause any detectable changes in neutrophil morphology (data not shown). These findings support the use of FSC-A as a reliable indicator of neutrophil shape change and activation, providing a simple, scalable approach to assess polarisation and morphological changes in response to chemoattractants or pharmacological modulators.

### Neutrophil activation induces forward scatter increases in different flow cytometer sorters and analysers

**Fig. 3:**
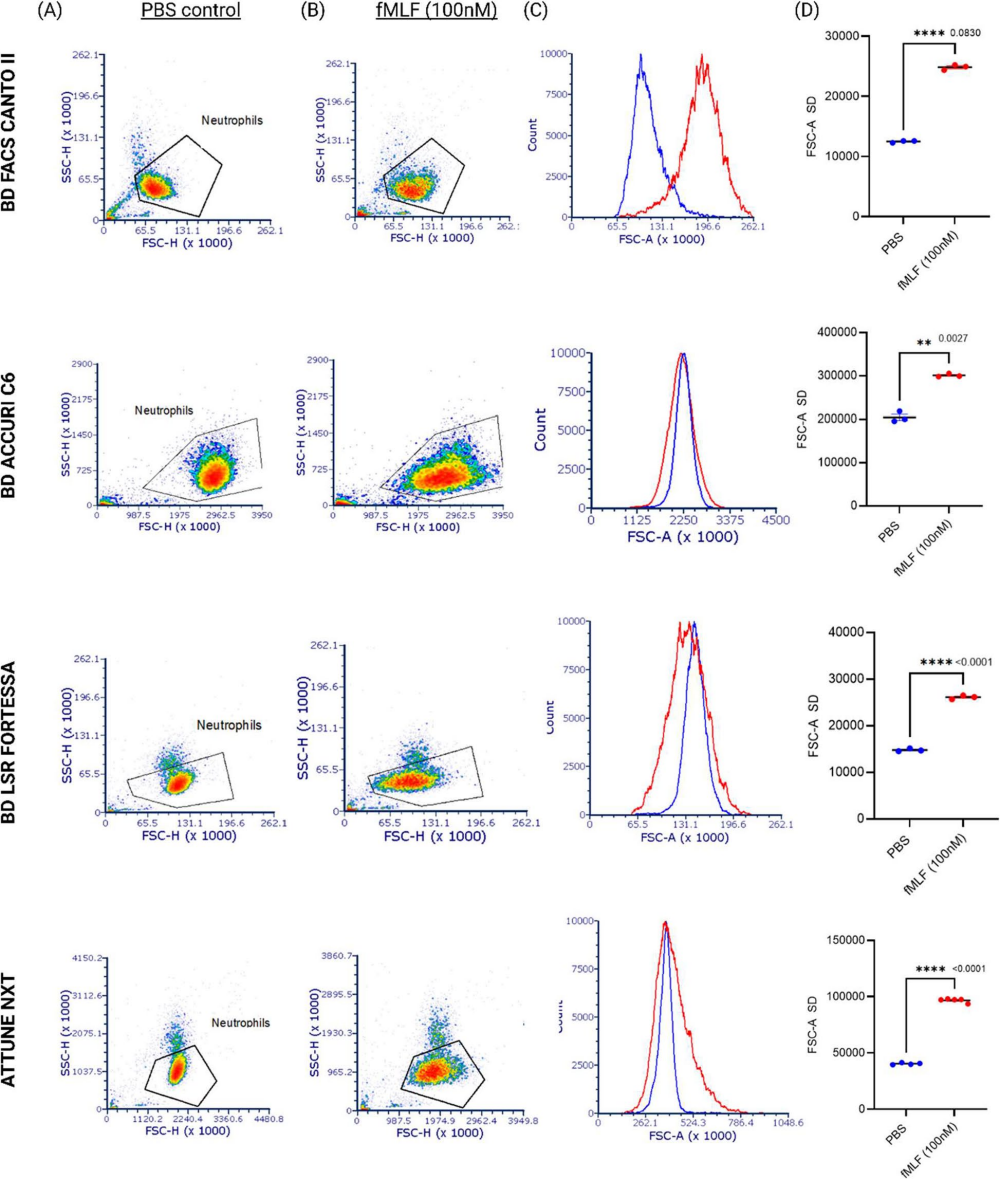
FSC profiles of human neutrophil shape change varies depending on the flow cytometry platform used. **A** Flow cytometry plots showing human neutrophils treated with PBS and analysed on different flow cytometry platforms (BD FACSCanto II, BD Accuri C6, BD LSR Fortessa, and Attune NXT). **B** Plots of neutrophils stimulated with 100 nM fMLF for 30 min show a rightward shift or broadening of the FSC-A histogram, depending on the instrument used. **C** Histogram overlays comparing PBS-treated (blue histogram) and fMLF-stimulated (red histogram) neutrophils highlight instrument-specific differences in FSC-A profiles, with sorters typically showing a rightward shift. **D** Quantitative analysis of neutrophil shape change based on the standard deviation of FSC-A (FSC-A SD) following PBS and fMLF treatment (*n* ≥ 3). **p ≤ 0.01, ****p ≤ 0.0001, unpaired t-test with Welch’s correction used to compare forward scatter between PBS control and fMLF-treated cells

Neutrophil shape-changes, reflected by increased forward light scatter (FSC-A), were detectable using flow cytometer sorters (e.g., BD FACS Aria II) and analysers (e.g., BD LSR Fortessa, Attune NXT). Forward versus side scatter plots from each instrument demonstrated consistent increases in FSC-A following 100 nM fMLF stimulation, indicating increased neutrophil shape and size alteration (Fig. 3A&B). However, differences in the distribution of FSC values on histogram plots were observed between instruments. This variability is influenced by the position and design of the obscuration bar, which in some analysers shifts the FSC histogram peak to the right, while others produce a wider distribution or shifts in the opposite direction (Fig. 3C).

**Fig. 4:**
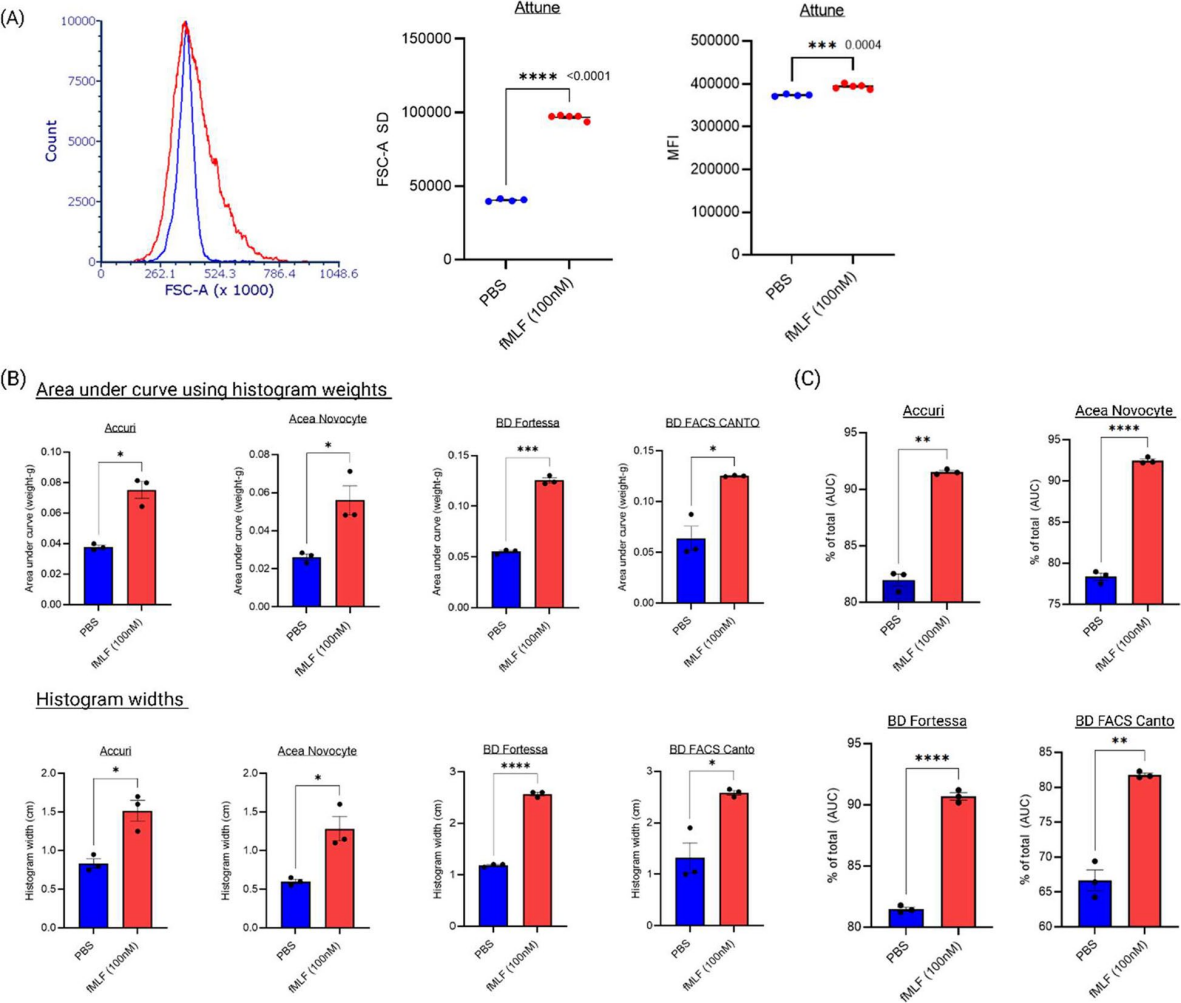
Neutrophil activation is measurable by physical histogram weighing and width analysis across multiple flow cytometers. **A** Graphical analysis of neutrophil activation by fMLF (100 nM) versus control using the Attune Nxt flow cytometer. Data are presented using histogram statistics, including mean fluorescent intensity (MFI) and standard deviation (SD) (*n* ≥ 4). **B** Alternative representation of activation data using histogram widths or area under the curve obtained by physically weighing carefully cut histograms. Data from multiple flow cytometers (as indicated in the figure) are shown for both control and fMLF-treated neutrophils for comparison (*n* = 3). **C** Activation data analysed using FCSExpress 7 flow cytometry software, showing the percentage of total cells based on a region of interest on the control and stimulated histograms. The area under the curve (AUC), depicted by the percentage of total cells within the defined region of interest, is shown. *p ≤ 0.05, ***p ≤ 0.001, ****p ≤ 0.0001; unpaired t-test with Welch’s correction comparing PBS control and fMLF-treated groups

Differences in data analysis between flow cytometry analysers and sorters, as well as the choice of statistical metrics, are important considerations when interpreting neutrophil shape change results. Mean fluorescent intensity (MFI) and forward scatter area standard deviation (FSC-A SD) are commonly used metrics to represent these changes. MFI reflects the fluorescent intensity of neutrophils within the singlet gate comparing stimulated versus unstimulated cells; however, its values vary depending on the instrument. For example, MFI increases with fMLF stimulation when measured on sorters where FSC-A histograms shift to the right, but decreases on some analysers like the LSR Fortessa, where FSC-A shifts to the left. Thus, MFI is relative and highly dependent on instrument settings and calibration. In contrast, FSC-A SD consistently provided more robust measurements of neutrophil shape change, accurately reflecting increases in cell size and morphological heterogeneity across the cell population. An increase in FSC-A SD was observed in fMLF-stimulated neutrophils compared to controls across all instruments tested (*n* = 5), supporting its use as a preferred metric for this assay (Figs. 3D and 4A). These findings thus demonstrate that both flow cytometry analysers and cell sorters can reliably detect stimuli-induced shape changes in cells, despite differences in scatter profiles due to instrumental design features.

Neutrophil shape change was also assessed using classical histogram-based methods, in which printed histograms of control and stimulated samples were physically cut, weighed, and their lateral width measured along the x-axis to compare distribution shifts [28]. Similarly, the histogram weights provide an estimate of the population shift in forward scatter, offering a simple yet effective method to compare activation-induced changes. These approaches produced results consistent with those obtained from flow cytometry sorters and analysers, showing clear distinctions between control and fMLF-stimulated neutrophils (Fig. 4B).

### Autofluorescence-based shape change analysis demonstrates eotaxin-specific eosinophil activation profiles

**Fig. 5:**
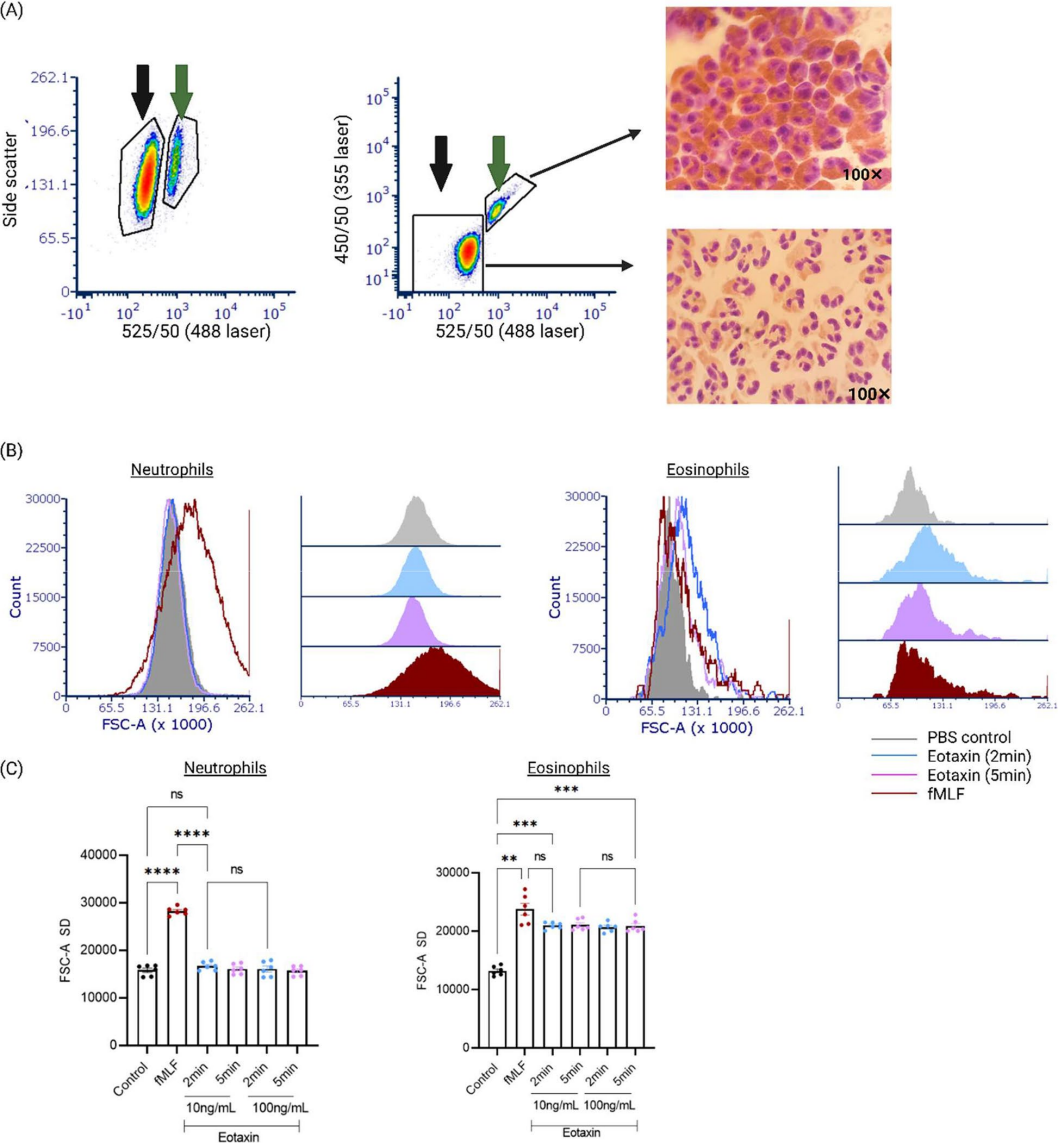
Eotaxin selectively triggers morphological changes in eosinophils but not neutrophils. **A** Human granulocytes isolated using the dextran/Percoll method were analysed by flow cytometry (using the Acea Novocyte flow cytometer), revealing a predominantly neutrophil population with minimal eosinophil contamination. Eosinophils (green arrow) were distinguishable from neutrophils (black arrow) based on their distinct autofluorescence. Representative micrographs of sorted neutrophils and eosinophils are shown. **B** Using the established gating strategy, the effect of human eotaxin on neutrophil and eosinophil shape change was assessed. Overlay histograms demonstrate that fMLF (100 nM) induces shape change in both cell types, while eotaxin (10 ng/mL) selectively activates eosinophils, with no effect on neutrophils. **C** Graphical summary of shape change responses in neutrophils and eosinophils treated with eotaxin (10 and 100 ng/mL) for 2 and 5 min, based on time-course experiments using cells from six independent donors (*n* = 6). *p ≤ 0.05, **p ≤ 0.01, ***p ≤ 0.001, ****p ≤ 0.0001; one-way ANOVA with Tukey’s multiple comparisons test was used to assess differences between treatment groups

To enable downstream assays, it was essential to isolate pure populations of neutrophils and eosinophils from mixed donor granulocyte preparations. Given the lack of specific surface markers to separate these two cell types without affecting their function, a previously described autofluorescence-based sorting strategy was used (20). This method considers the intrinsic differences in autofluorescent properties between neutrophils and eosinophils under specific flow cytometry laser settings. Clear separation between neutrophils and eosinophils was achieved based on their autofluorescence profiles in the 488 nm (525/50 nm) and 355 nm (450/50 nm) detectors. In addition, cytocentrifuge preparations of sorted cells from the respective gates confirmed high purity of both cell populations, as verified by H&E (Haematoxylin and Eosin) staining (Fig. 5A).

To evaluate the effect of human eotaxin on donor-derived neutrophils and eosinophils, forward scatter-based shape change assays were performed. Compared to fMLF, which robustly activated neutrophils, eotaxin selectively induced shape change in eosinophils but not neutrophils at concentrations of 10 and 100 ng/mL (*n* = 6) (Fig. 5B and C). These findings highlight the use of forward scatter analysis for studying selective immune cell activation in response to specific agonists. Additionally, time-course experiments (data not shown) and histogram profiles of forward scatter data showed that eotaxin-mediated eosinophil activation occurs rapidly, within 2–5 min, whereas fMLF-induced neutrophil activation was observed at approximately 30 min (Fig. 5B and C).

### 5-oxo-ETE induces rapid and reversible shape-change in both neutrophils and eosinophils via forward-scatter profiling

To further evaluate the use of this method in assessing granulocyte activation, we investigated the effects of additional lipid mediators on neutrophil and eosinophil shape change. Using forward scatter (FSC-A) profiles as a readout, we compared the activation potential of 5-oxo-ETE, a known eicosanoid associated with inflammation, to that of fMLF and eotaxin. This method allowed us to determine the relative specificity and activation kinetics in both human neutrophils and eosinophil populations.

**Fig. 6:**
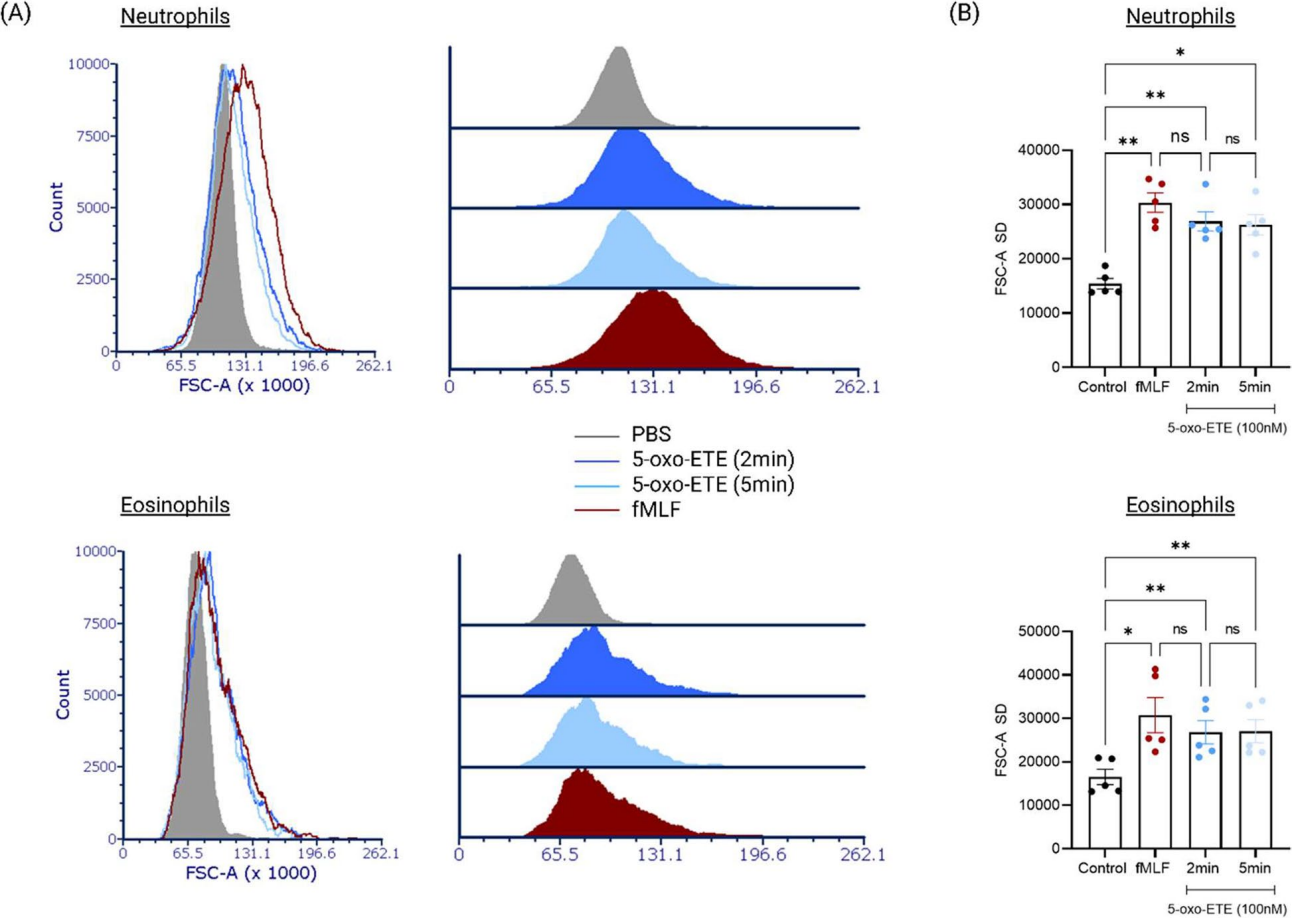
5-oxo-eicosatetraenoic acid induces shape change in both human neutrophils and eosinophils in vitro. **A** Flow cytometry (using the Acea Novocyte flow cytometer) analysis shows that 5-oxo-ETE (100 nM), a lipid mediator, induces rapid shape change in human eosinophils, with peak activation at 2 min post-stimulation. The observed response is comparable to that seen with fMLF (100 nM). In neutrophils, 5-oxo-ETE also triggers shape change, evident by increased forward scatter, though the effect is less than that induced by fMLF. **B** Quantitative representation of shape change in neutrophils and eosinophils, following stimulation with 100 nM 5-oxo-ETE for 2 and 5 min, based on time-course experiments using cells from five individual donors (*n* = 5). *p ≤ 0.05, **p ≤ 0.01; one-way ANOVA with Tukey’s multiple comparisons test comparing treatment groups

5-oxo-ETE was evaluated for its ability to induce shape change in human granulocytes. At a concentration of 100 nM, it triggered forward scatter increases in both neutrophils and eosinophils, indicating cellular activation (Fig. 6A). While the response in eosinophils was comparable to that seen with fMLF, neutrophils showed a weaker response relative to fMLF stimulation. Similar to eotaxin, 5-oxo-ETE induced a rapid and transient shape change response in both cell types, peaking within 2 min and declining by 5 min (*n* = 5) (Fig. 6B).

## Discussion

This study represents a simple, rapid and scalable method to reliably detect granulocyte activation in response to inflammatory stimuli, through changes in cell morphology, using forward scatter (FSC-A) and autofluorescence properties by flow cytometry. Our findings support the use of FSC-A shifts as a readout for both neutrophil and eosinophil activation, providing a label-free method to detect shape changes in these immune cells. During an infection or sterile injury, neutrophil activation occurs when pathogen and damage-derived formylated peptides bind the G-protein coupled receptor FPR1, triggering calcium flux and downstream functions such as chemotaxis, phagocytosis, degranulation, ROS production and formation of extracellular traps [4, 33]. While these responses aid host defence, dysregulated activation contributes to various inflammatory diseases. Therefore, targeting receptor signalling through modulation of GPCR signalling to limit immune cell activation could offer therapeutic benefit. The synthetic peptide, fMLF, used in this study is a well-established FPR1 agonist and is often used to model neutrophil activation in vitro [4, 33]. Similarly, lipid mediators like LTB_4_ can activate neutrophils via the GPCR BLT1, and formylated peptides can also act as priming agents to promote release of LTB_4_ and IL-8, to further amplify the inflammatory response [6]. Although previous studies have examined the role of LTB_4_ and its receptor antagonist in neutrophil shape change and apoptosis, comparative analyses with other receptor agonists and antagonists, such as CsH, have not been reported. Our study addresses this gap by directly comparing these effects and moreover, while morphological changes in primed and activated neutrophils have been described, we employ imaging flow cytometry to visualise these changes in real-time, offering new insights into the dynamic behaviour of neutrophils with detail not previously achieved.

One of the main advantages of this approach is its adaptability across different flow cytometry instruments. Although the shape and size of FSC-A histograms vary between analysers and sorters used, we have successfully shown that the overall outcome in shape change remains consistent across different instruments. The variability in FSC-A histograms is due to the position and design of the obscuration bar, which in some analysers shift the FSC peak to the right, while in others produce a wider distribution or shift in the opposite direction. The obscuration bar, positioned in front of the FSC detection lens, reduces stray light by blocking unwanted scatter, allowing only light scattered at specific angles to reach the FSC detector [34]. This enhances FSC resolution by minimizing background noise. Therefore, this work highlights the robustness of the assay, while also emphasising the importance of using standardised statistical parameters such as FSC-A SD instead of MFI (based on instrument settings and calibration), to account for differences in instrument calibration and alignment.

In addition, we confirm previous findings that granulocyte populations, in particular neutrophils and eosinophils can easily be distinguished using their inherent autofluorescence properties by flow cytometry. This antibody-free approach allows clean separation between the cell types using intrinsic cellular autofluorescence properties induced by the 488 nm and 355 nm lasers, without the need for surface staining, which was further validated using H&E stains of sorted cells. In addition, this method can be applied using even the most basic flow cytometers by relying on side scatter profiles when the ultraviolet laser is not available. This approach is further useful for assessing functional responses, where antibody binding could potentially influence cell behaviour. Fixation was carried out only when long-term storage was required; otherwise, unfixed cells were used immediately and could be maintained on ice for 10–30 min, as previously described, to preserve cellular integrity [20].

To this end, our results show that both fMLF and 5-oxo-ETE induce shape change or morphological activation in neutrophils and eosinophils, while eotaxin selectively activates eosinophils which is consistent with published receptor biology, where fMLF acts via FPR1/FPR2, 5-oxo-ETE via OXER1, and eotaxin via CCR3, expressed on eosinophils [4, 35, 36]. Interestingly, the rapid kinetics observed with both 5-oxo-ETE and eotaxin further indicates that this assay is suitable for assessing early activation of immune cells.

For the first time, the data also show that granulocyte responses to 5-oxo-ETE and eotaxin are transient, peaking between 2 and 5 min and declining thereafter. This pattern is consistent with the in vivo granulocyte behaviour, where shape change precedes transmigration to inflammatory sites [37]. Interestingly, eotaxin acts a negative regulator for neutrophil activation and recruitment, in response to inflammatory challenges either through altering their responsiveness to cytokines or reducing adhesion and transmigration, as seen in a mouse model of endotoxemia [38]. In addition, eosinophil activation with eotaxin has previously been shown to enhance neutrophil responses through LTB_4_ and 5-oxo-ETE production via 5-lipoxygenase (5-LOX) pathway, thereby suggesting a possible feedback mechanism [9, 39]. This further highlights a role for eosinophils in secondary neutrophil recruitment.

The speed, scalability, cost-effectiveness, and simplicity combined with a framework suitable for high-throughput screening make this method highly suitable for early-stage drug discovery in models of inflammatory disease. However, certain limitations of the method should be acknowledged. Forward scatter data are essentially dependent on instrument optics, and autofluorescence gating of eosinophils and neutrophils may be influenced by differences in granule content in disease states. While shape change is a strong indicator of activation, it does not incorporate all aspects of granulocyte functional responses such as ROS production, degranulation, or cytokine release. Hence, it is a valuable technique that should be combined with complementary assays for in-depth assessment.

In brief, this study incorporates a rapid, robust, and versatile method for detecting activation-induced shape changes in granulocytes. Furthermore, this technique provides valuable insights into immune cell behaviour and a practical tool for mechanistic studies and drug screening.

## Conclusions

This study presents a robust, scalable and label-free methodology to assess changes in granulocyte activation through shape-change analysis in response to different agonists and antagonists using scatter and autofluorescent properties. The approach allows effective distinction between neutrophil and eosinophil responses to the same agonist, offering a valuable tool for screening pharmacological agents targeting immune cell activation. Our Findings reveal that eotaxin selectively activates eosinophils, but not neutrophils, while 5-oxo-ETE induces shape change in both cell types, highlighting their distinctive responsiveness. Interestingly both agonists trigger rapid and reversible activation, indicating their relevance in acute inflammatory processes. Overall, the method provides a platform for studying granulocyte biology and identifying candidate therapeutics to mitigate immune-driven inflammation.

## Supplementary Information

Supplementary Material 1: Supplementary Figure 1. Concentration- and time-dependent effects of GPCR modulators on human neutrophil shape change. (A) An initial concentration-response curve for fMLF (an FPR1 agonist) on human neutrophils from one donor, identifying 100nM as an appropriate test concentration for subsequent experiments. (B) Time-course analysis indicating that a 30min pre-incubation with 10µM CsH is sufficient to inhibit the fMLF-induced shape change response (*n*=2). (C) Concentration-response and (D) time-course analyses were performed using LTB_4_ (1nM and 100nM) to identify the optimal concentration and incubation period for BLT1 activation. The conditions selected for subsequent experiments is indicated with a red asterix (*). All above experiments were performed on the BD FACSAria II flow cytometer and analysed using the FCSExpress 7 (research edition) software program. Supplementary Figure 2. 5-oxo-ETE triggers rapid, concentration-dependent activation of both human neutrophils and eosinophils. (A) Time-course data of neutrophil (left) and eosinophil (right) shape change following stimulation with 5-oxo-ETE at 1nM, 10nM and 100nM doses, quantified using the FSC-A SD with measurements taken from 2 to 30 minutes post-stimulation using the Acea Novocyte flow cytometer. A rapid, concentration-dependent increase in shape change was observed in both cell types at early time points. (B) Representative flow cytometry histograms showing FSC-A shifts in human eosinophils stimulated for 2min with PBS (black), 100nM fMLF (red), or 100nM 5-oxo-ETE (blue) indicating a clear increase in shape change with 5-oxo-ETE stimulation. (C) FSC-A histogram plots of eosinophils following 2 minutes stimulation with 1nM, 10nM, and 100nM 5-oxo-ETE, depicting a concentration-dependent shift in FSC-A consistent with shape change. Supplementary Figure 3. fMLF stimulation of human neutrophils induces alterations in cell surface marker expression and ROS production. (A) Flow cytometry gating strategy identifying viable human neutrophils based on forward and side scatter, singlets and exclusion of dead cells using DAPI staining. Cells were further gated for CD62L and CD11b expression (control., CD62L^high^/CD11b^low^ and fMLF-stimulated., CD62L^low^/CD11b^high^). (B) Flow cytometry histograms and quantification showing loss of CD62L and upregulation of CD11b following fMLF stimulation (red histogram) (*n*=3). (C) Quantification of DHR1,2,3 fluorescence by flow cytometry showing increased intracellular ROS production in neutrophils stimulated with 100nM fMLF compared to control (*n*=3). *p ≤0.05, ****p ≤0.0001; unpaired t-test with Welch’s correction was used to compare cell surface marker expression and ROS levels between control and fMLF-stimulated neutrophils.

## Data Availability

Data supporting the findings of this study are available in the supplementary section and from the corresponding author upon reasonable request. Reagents and materials used in the study can also be provided upon request, subject to material transfer agreements where applicable.

## Abbreviations

fMLF: N-formylmethionine-leucyl-phenylalanine
LTB_4_: Leukotriene B_4_
IL-8: Interleukin-8
ROS: Reactive oxygen species
FSC: Forward scatter
SSC: Side scatter
GPCR: G-protein coupled receptor
5-oxo-ETE: 5-Oxo-eicosatetraenoic acid
