# Supplementary material for: Rapid autofluorescence flow cytometric analysis of agonist-induced neutrophil and eosinophil polarization reveals novel insights into 5-oxo-ETE-mediated granulocyte activation
Source: J Inflamm (Lond). 2025 Nov 6;22:47. doi: 10.1186/s12950-025-00472-8 (PMC12593794; doi:10.1186/s12950-025-00472-8)
Supplement: Supplementary file 1 — Supplementary Material 1: Supplementary Figure 1. Concentration- and time-dependent effects of GPCR modulators on human neutrophil shape change. (A) An initial concentration-response curve for fMLF (an FPR1 agonist) on human neutrophils from one donor, identifying 100nM as an appropriate test concentration for subsequent experiments. (B) Time-course analysis indicating that a 30min pre-incubation with 10µM CsH is sufficient to inhibit the fMLF-induced shape change response (n=2). (C) Concentration-response and (D) time-course analyses were performed using LTB4 (1nM and 100nM) to identify the optimal concentration and incubation period for BLT1 activation. The conditions selected for subsequent experiments is indicated with a red asterix (*). All above experiments were performed on the BD FACSAria II flow cytometer and analysed using the FCSExpress 7 (research edition) software program. Supplementary Figure 2. 5-oxo-ETE triggers rapid, concentration-dependent activation of both human neutrophils and eosinophils. (A) Time-course data of neutrophil (left) and eosinophil (right) shape change following stimulation with 5-oxo-ETE at 1nM, 10nM and 100nM doses, quantified using the FSC-A SD with measurements taken from 2 to 30 minutes post-stimulation using the Acea Novocyte flow cytometer. A rapid, concentration-dependent increase in shape change was observed in both cell types at early time points. (B) Representative flow cytometry histograms showing FSC-A shifts in human eosinophils stimulated for 2min with PBS (black), 100nM fMLF (red), or 100nM 5-oxo-ETE (blue) indicating a clear increase in shape change with 5-oxo-ETE stimulation. (C) FSC-A histogram plots of eosinophils following 2 minutes stimulation with 1nM, 10nM, and 100nM 5-oxo-ETE, depicting a concentration-dependent shift in FSC-A consistent with shape change. Supplementary Figure 3. fMLF stimulation of human neutrophils induces alterations in cell surface marker expression and ROS production. (A) Flow cytomet [file 12950_2025_472_MOESM1_ESM.docx]

**Supplementary Data: Figures 1, 2 and 3**

**
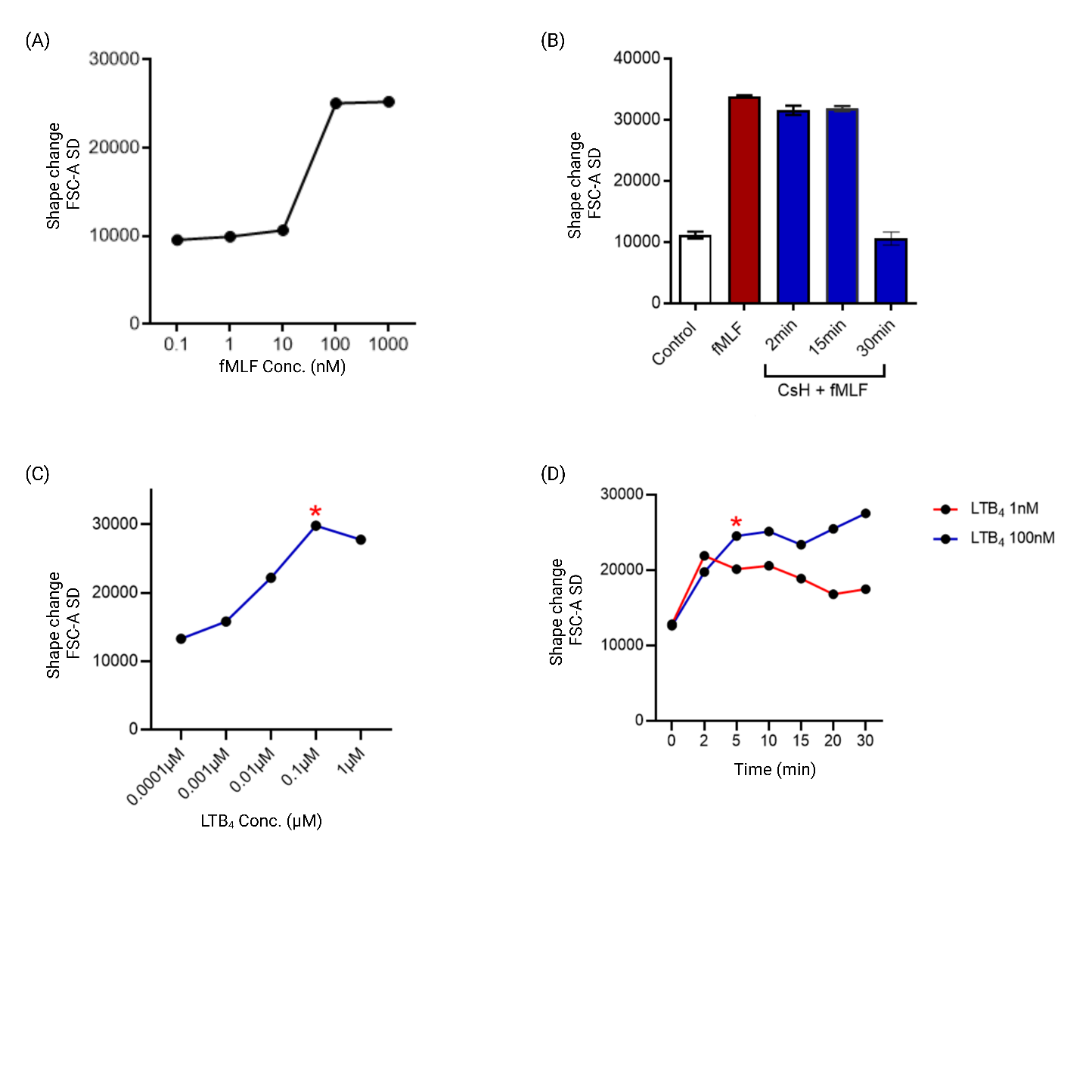
**

**Supplementary Figure 1: Concentration- and time-dependent effects of GPCR modulators on human neutrophil shape change.** (A) An initial concentration-response curve for fMLF (an FPR1 agonist) on human neutrophils from one donor, identifying 100nM as an appropriate test concentration for subsequent experiments. (B) Time-course analysis indicating that a 30min pre-incubation with 10µM CsH is sufficient to inhibit the fMLF-induced shape change response (n=2). (C) Concentration-response and (C) time-course analyses were performed using LTB_4_ (1nM and 100nM) to identify the optimal concentration and incubation period for BLT1 activation. The conditions selected for subsequent experiments is indicated with a red asterix (*). All above experiments were performed on the BD FACSAria II flow cytometer and analysed using the FCSExpress 7 (research edition) software program.

**
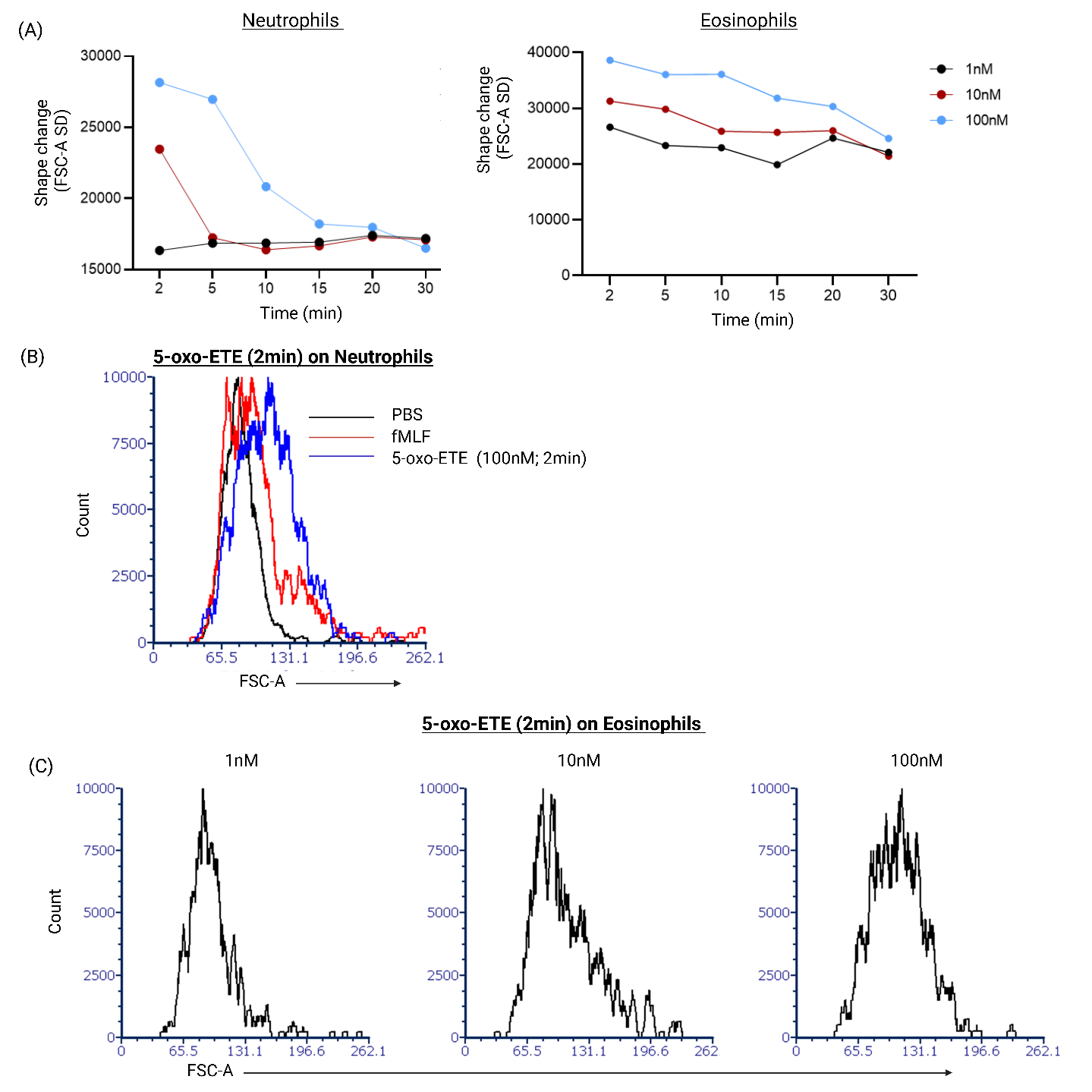
**

**Supplementary Figure 2: 5-oxo-ETE triggers rapid, concentration-dependent activation of both human neutrophils and eosinophils.** (A) Time-course data of neutrophil (left) and eosinophil (right) shape change following stimulation with 5-oxo-ETE at 1nM, 10nM and 100nM doses, quantified using the FSC-A SD with measurements taken from 2 to 30 minutes post-stimulation using the Acea Novocyte flow cytometer. A rapid, concentration-dependent increase in shape change was observed in both cell types at early time points. (B) Representative flow cytometry histograms showing FSC-A shifts in human eosinophils stimulated for 2min with PBS (black), 100nM fMLF (red), or 100nM 5-oxo-ETE (blue) indicating a clear increase in shape change with 5-oxo-ETE stimulation. (C) FSC-A histogram plots of eosinophils following 2 minutes stimulation with 1nM, 10nM, and 100nM 5-oxo-ETE, depicting a concentration-dependent shift in FSC-A consistent with shape change.

**
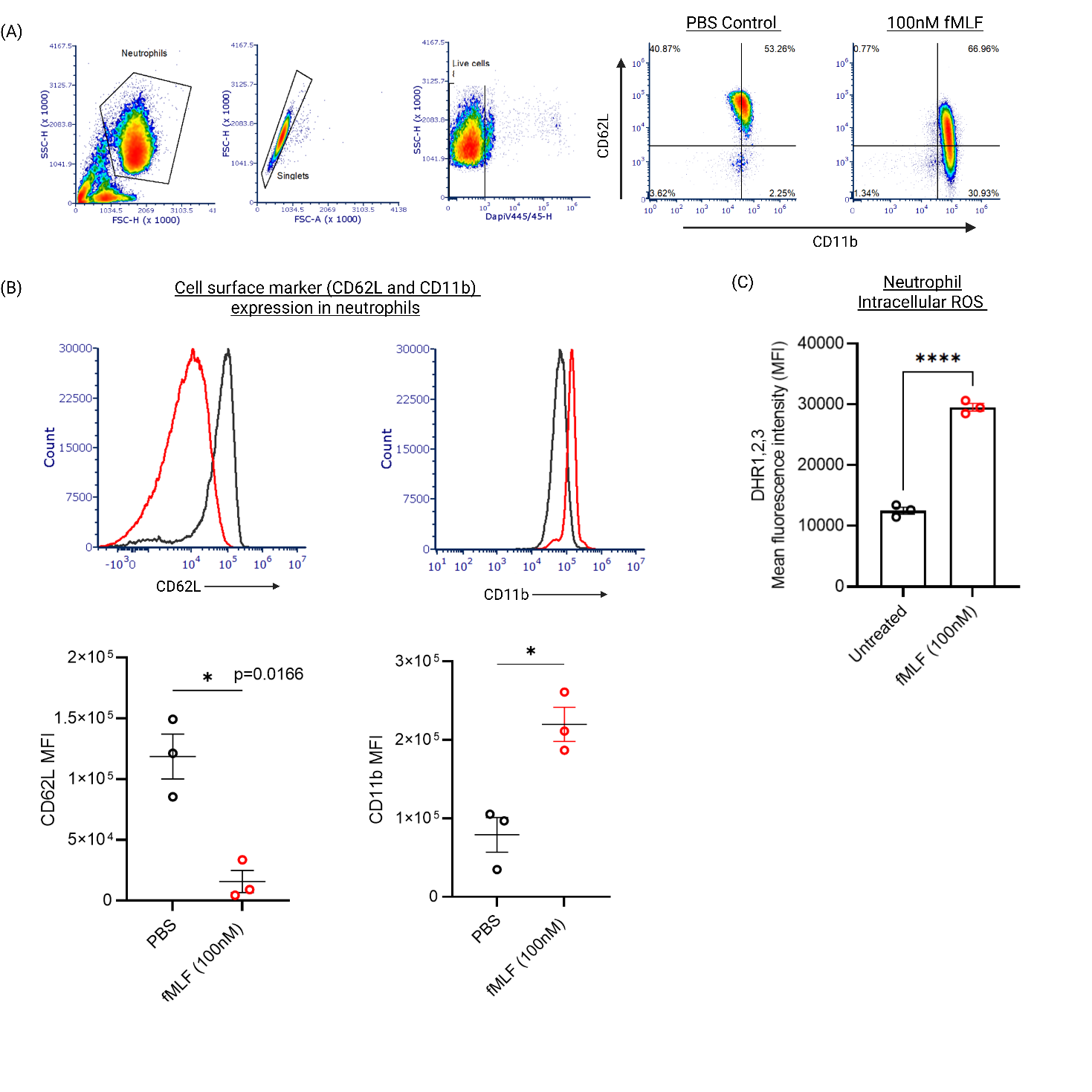
**

**Supplementary Figure 3: fMLF stimulation of human neutrophils induces alterations in cell surface marker expression and ROS production.** (A) Flow cytometry gating strategy identifying viable human neutrophils based on forward and side scatter, singlets and exclusion of dead cells using DAPI staining. Cells were further gated for CD62L and CD11b expression (control., CD62L^high^/CD11b^low^ and fMLF-stimulated., CD62L^low^/CD11b^high^). (B) Flow cytometry histograms and quantification showing loss of CD62L and upregulation of CD11b following fMLF stimulation (red histogram) (n=3). (C) Quantification of DHR1,2,3 fluorescence by flow cytometry showing increased intracellular ROS production in neutrophils stimulated with 100nM fMLF compared to control (n=3). *p≤0.05, ****p≤0.0001; unpaired t-test with Welch’s correction was used to compare cell surface marker expression and ROS levels between control and fMLF-stimulated neutrophils.
